# Usefulness of continuous probability distributions of rates for modelling radionuclide biokinetics in humans and animals

**DOI:** 10.1038/s41598-018-38046-9

**Published:** 2019-02-04

**Authors:** Igor Shuryak, Ekaterina Dadachova

**Affiliations:** 1 Center for Radiological Research, Columbia University, New York, NY USA; 2 University of Saskatchewan, Saskatoon, SK Canada

## Abstract

Modelling the biokinetics of radionuclide excretion or retention is important in nuclear medicine and following accidental/malicious radioactivity releases. Sums of discrete exponential decay rates are often used, but we hypothesized that continuous probability distributions (CPD) of decay rates can describe the data more parsimoniously and robustly. We tested this hypothesis on diverse human and animal data sets involving various radionuclides (including plutonium, strontium, caesium) measured in the laboratory and in regions contaminated by the Fukushima and Chernobyl nuclear accidents. We used four models on each data set: mono-exponential (ME) with one discrete decay rate, bi-exponential (BE) with two rates, gamma-exponential (GE) with a Gamma distribution of stretched-exponential rates, and power-decay (PD) with a Gamma distribution of power-decay rates. Information-theoretic model selection suggested that radionuclide biokinetics, e.g. for plutonium in humans, are often better described by CPD models like GE and PD, than by discrete rates (ME and BE). Extrapolation of models fitted to data at short times to longer times was frequently more robust for CPD formalisms. We suggest that using a set of several CPD and discrete-rate models, and comparing them by information-theoretic methods, is a promising strategy to enhance the analysis of radionuclide excretion and retention kinetics.

## Introduction

Mathematical models of radionuclide excretion and retention kinetics from living organisms are important in a variety of contexts. For example, they are needed to estimate radiation doses and health risks from medical (e.g. nuclear medicine procedures), accidental (e.g. nuclear power plant accidents like Chernobyl or Fukushima), malicious (e.g. terrorist attacks using radioactive materials) or occupational (e.g. nuclear industry workers) exposures resulting in radionuclide dispersal and/or incorporation into the body. Such models are also needed in application to organisms other than humans, e.g. when nuclear power plant accidents such as Fukushima cause radioactive contamination of fish and game animals that are used for human consumption.

Radionuclides undergo well-understood physical decay. Importantly, however, many other chemical, biological and ecological processes also affect the kinetics of their removal from living organisms. Whereas physical decay has an exponential time dependence, these other processes can be much more complex and result in non-exponential time patterns. Detailed models have been developed to address this complexity, e.g. human radionuclide biokinetics models^1^ and models for the uptake and turnover of radionuclides in ecosystems^2^.

Complex models, however, have some important limitations. When the number of modelled processes that operate on different time scales and often have non-linear dependences is large, and the number of model parameters is correspondingly large, the model can become difficult to solve and parameter estimates can have very large uncertainties^3,4^. The latter phenomenon is sometimes called model “sloppiness”^4^.

Here we investigated the possibilities of using simple models, with small numbers of adjustable parameters, to describe radionuclide biokinetics data. For this purpose, we developed two new simple models based on the concept of a continuous probability distribution (CPD) of decay rates. The first, abbreviated as gamma-exponential (GE), combined the stretched exponential function^5^ with a Gamma distribution of rates. The second, abbreviated as power-decay (PD), combined a simplified version of the stretched hyperbola with a Gamma distribution of rates^6^. Using the Akaike information criterion with sample size correction (AICc) and multimodel inference (MMI)^7,8^, which are described in the Methods section, we compared the performances of these models with those of the commonly-used mono-exponential (ME) and bi-exponential (BE) models^9–11^, which represent a single decay rate and the sum of two rates, respectively.

For the comparisons, we used the following diverse real data sets, both human and animal, as examples. (I) Urinary excretion of plutonium in healthy human volunteers over time after administration^12^. (II) Plasma concentrations of strontium in healthy human volunteers over time after administration^13^. (III). Animal data measured under laboratory conditions: (a) Concentrations of humanized melanin-binding ^111^In-labeled IgG antibodies in mouse blood over time after injection. (b) ^137^Cs retention in the sea urchin *Strongylocentrotus nudus*^14^. (IV). Caesium (^137^Cs, ^134^Cs) radioactivity concentrations in the following animals inhabiting the area contaminated by the Fukushima nuclear power plant accident in Japan: (a) wild boars (*Sus scrofa*), (b) Asian black bears (*Ursus thibetanus*), (c) sika deer (*Cervus nippon*), and (d) ocellate spot skate (*Okamejei kenojei*). (V). ^137^Cs radioactivity concentrations in wild boars (*Sus scrofa*) in the area contaminated by the Chernobyl nuclear power plant accident in Ukraine^15^.

The main goal of this study was to conduct a proof of principle investigation of whether or not continuous probability distribution models like GE and PD could be reasonable for application in the fields of radionuclide biokinetics and radioecology, compared with models with discrete rates like ME and BE. The concept of continuous rate distribution models would be potentially useful to investigators in these fields because different types of biological and/or ecological processes can be summarized, producing robust numerical predictions, by continuous distributions of rates. In situations when the kinetics data for a particular radionuclide are well fitted by GE or PD functions, but not by ME or BE functions, the result suggests that many (rather than only 1 or 2) compartments and/or rates are likely to be involved. Detailed mechanistic models (*e.g*.)^1^ would be needed to refine the information on specific rates and compartments, but such models may not be available for some studied radionuclides and/or organisms.

## Methods

### Data sets

Our goal was to apply the proposed set of models (ME, BE, GE and PD) to a diverse set of real data examples on radionuclide biokinetics from both humans and animals to assess a realistic range of model performance patterns, e.g. dominance of discrete rate models, dominance of CPD models, or no clearly best-supported model(s). For this purpose, we identified the following data sets.

(I) Urinary excretion of plutonium in healthy human volunteers over time after administration^12^. Each study subject ingested ^244^Pu citrate solution and was injected intravenously with additional ^244^Pu several months later. This type of administration mimics a complicated pattern of plutonium incorporation into the human body that can result from accidental or malicious radioactive contamination. Here we analysed the urinary excretion data on 5 male volunteers over time after injection, which were presented in Table 4 of Ham *et al*.^12^. On this and all other data sets analysed here, we ln-transformed the data to bring the uncertainty distribution closer to Normal. We analysed the data from each subject separately, instead of resorting to mixed-effects modelling, because we focused on comparing the performances of different models by information-theoretic methods (described below), rather than on variations in parameter values for a single model across subjects.
(II) Plasma concentrations of strontium in healthy human volunteers over time after administration^13^. Each study subject ingested ^86^Sr and was simultaneously injected intravenously with ^84^Sr. We analysed the data on 3 volunteers (separately for each one), which were presented in Table 4 of Li *et al*.^13^.
(III) Animal data measured under laboratory conditions. The first example involves a vertebrate model organism, the C57Bl6 female laboratory mouse. We used our own unpublished data on the kinetics of ^111^In-labeled IgG antibody to melanin in mouse blood over time after injection, corrected for physical decay of ^111^In. The data are presented in Supplementary Data File 1. The second example involves an invertebrate model organism, the sea urchin *Strongylocentrotus nudus*^14^. We analysed the data on ^137^Cs excretion in sea urchins, which were obtained by first holding the urchins in radioactively contaminated water for 7 days, and then transferring them to clean water and monitoring their radioactivity levels over time. These data were presented in Fig. 1 of Nakamura *et al*.^14^, where each data point is the average value for 5 urchins. We digitized the data using GetData Graph Digitizer 2.26 software (http://getdata-graph-digitizer.com/).

**Figure 1:**
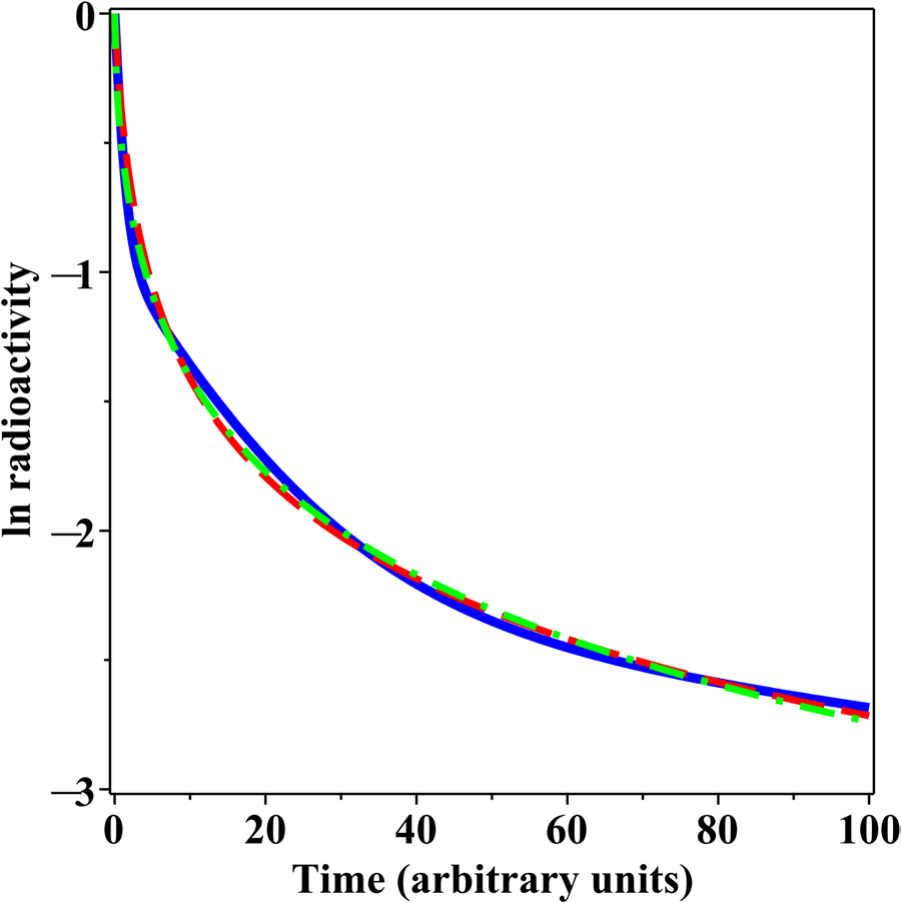
Comparison of decay patterns generated by a sum of three exponential rates (solid blue curve) and by the GE model (green dashed curve) and the PD model (red dashed curve) fitted to these patterns. Details are described in the main text.
(IV) Radiocaesium (combined ^137^Cs and ^134^Cs) radioactivity concentrations in wild game animals inhabiting the area contaminated by the Fukushima nuclear power plant accident in Japan: (a) wild boars (*Sus scrofa*), (b) Asian black bears (*Ursus thibetanus*), and (c) sika deer (*Cervus nippon*) (Supplementary Data File 2). These data were obtained from the Japan Atomic Energy Agency (JAEA) Radiation Monitoring Survey Results of Wild Birds and Animals (https://emdb.jaea.go.jp/emdb/en/portals/1040501000/). We analysed the data collected after September 1, 2011, i.e. approximately 6 months after the accident, because by this time the accumulation of radionuclides within animals probably reached its full extent and their subsequent excretion kinetics could be evaluated. This time frame was chosen because earlier times would likely be too soon for the ecological factors of radionuclide uptake and excretion in animals inhabiting the contaminated zone to reach a semi-equilibrium. Instances when the radiocaesium level was below detection (usually <10 Bq/kg) were rare (0.8% of observations for wild boars, 4.1% for black bears, and 6.1% for sika deer), and we excluded them from analysis. The exclusion of these small fractions of very low radioactivity measurements should not substantially bias the analysis results.
  Since only the sum of ^137^Cs and ^134^Cs activities, rather than each isotope individually, was recorded in animal tissues, we estimated the ratio of these two isotopes using data from the JAEA Unmanned Helicopter Monitoring in the Distribution Survey of Radioactive Substances (https://emdb.jaea.go.jp/emdb/en/portals/b1020202/). We assumed that the isotope ratio would be the same for all studied animals, and estimated it to be about 47.4% ^134^Cs vs 52.6% ^137^Cs on September 1, 2011. The ratio changed at subsequent times based on the physical half-lives of ^134^Cs and ^137^Cs.
  In addition to these data on terrestrial mammals, we also analysed data on ^137^Cs in a bottom-dwelling marine fish, the ocellate spot skate (*Okamejei kenojei*) (Supplementary Data File 2). We used data collected in Fukushima prefecture, starting on April 1, 2012 (no earlier measurements were available), taken from the Results of the monitoring on radioactivity level in fisheries products database (http://www.jfa.maff.go.jp/e/inspection/). Because the fraction of instances with undetectable ^137^Cs levels increased over time after the accident, the end date of May 13, 2015 was chosen to keep this fraction under 10%, so that exclusion of undetectable measurements from analysis would not substantially bias the results.
(V) ^137^Cs radioactivity concentrations in wild boars (*Sus scrofa*) in the area contaminated by the Chernobyl nuclear power plant accident in Ukraine. These data were presented in Table 1 of Gulakov^15^. We used time since 1991, in years, as the independent variable for this analysis.

**Table 1:** Comparison of model performances on different data sets.

Data sets: organisms, radionuclides		Best-supported model (the one with lowest AICc)	Sum of Akaike weights for GE and PD models	Minimum RMSE loss for	
				GE and PD models	ME and BE models
I. Humans, plutonium	Subject 1	**PD**	**1.000**	**110.3**	7706.3
	Subject 2	**PD**	**1.000**	**19.3**	7367.0
	Subject 3	**PD**	**1.000**	**−61.6**	2246.0
	Subject 4	BE	0.379	**1037.2**	144556.7
	Subject 5	**PD**	**1.000**	**166.7**	28807.9
II. Humans, strontium	Subject 1	ME	0.272	**406.7**	1002.1
	Subject 2	**GE**	**0.667**	**248.8**	4543.5
	Subject 3	ME	0.002	**838.5**	1132.7
III. Laboratory animals	Mouse	ME	0.493	**42.4**	67.6
	Sea urchin	BE	**0.549**	**273.5**	729.4
IV. Wild animals in Fukushima nuclear accident zone, caesium	Wild boar	**GE**	**0.943**	**26.6**	50.3
	Black bear	ME	0.245	107.8	**106.7**
	Sika deer	BE	0.277	48.9	**−23.9**
	Ocellate spot skate	BE	**0.533**	−121.5	**−132.1**
V. Wild animals in Chernobyl nuclear accident zone, caesium	Wild boar	NA	NA	NA	NA

Instances when CPD models were favoured over discrete rate models are shown in bold font. The NA label for data set V indicates that these data were explained exclusively by physical decay of radionuclides, as described in the main text, and all tested models therefore produced equivalent fits.

Because the focus of the current study is to investigate the *time* dependence of radioactivity excretion or retention processes, in all of these data sets we did not include other variables such as location. The effects of these other variables were not explicitly modelled here and were treated as components of random noise.

### Models

The simplest mono-exponential (ME) decay model is represented by the following equation, where *R*_*ME*_ (*t*) is the radioactivity at time *t*, *Q* is the intercept parameter (exp[*Q*] is the radioactivity at *t* = 0), *PD*(*t*) is the physical decay function of the radioactivity, and *k* represents other radioactivity excretion and retention processes (e.g. biochemical, ecological):

1 $${R}_{ME}(t)=\exp [Q]\times \exp [\,-\,k\times t]\times PD(t)$$

On a logarithmic scale, equation 1 can be rewritten as follows:

2 $$\mathrm{ln}\,[{R}_{ME}(t)]=Q+\,\mathrm{ln}\,[PD(t)]-k\times t$$

The physical decay function *PD*(*t*) for *X* radionuclides is the following sum of exponential dependences, where *C*_*j*_ is the fractional contribution of the *j-*th radionuclide at *t* = 0, and *Th*_*j*_ is the physical half-life of this radionuclide:

3 $$PD(t)=\sum _{j=1}^{X}\,{C}_{j}\times \exp \,[-\,\mathrm{ln}[2]\times \frac{t}{T{h}_{j}}]$$

The bi-exponential (BE) decay model is represented by the following equation, where *Q* and *PD*(*t*) have the same meanings as in the ME model, *k*_2_ is the slow radioactivity reduction process, and *k*_1_ is the additional reduction process, which acts on fraction *F* of the radioactivity:

4 $${R}_{BE}(t)=\exp [Q]\times (F\times \exp [-({k}_{1}+{k}_{2})\times t]+(1-F)\times \exp [-\,{k}_{2}\times t])\times PD(t)$$

The parametrization of equation 4 was chosen so that when *k*_1_ ≥ 0 and *k*_2_ ≥ 0, then *k*_1_ + *k*_2_ ≥ *k*_2_, thereby unambiguously defining *F* as the “fast-decaying” fraction of radioactivity and 1 – *F* as the “slow-decaying” fraction. On a logarithmic scale, equation 4 can be rewritten as follows:

5 $$\mathrm{ln}\,[{R}_{BE}(t)]=Q+\,\mathrm{ln}\,[PD(t)]+\,\mathrm{ln}\,[F\times \exp [-({k}_{1}+{k}_{2})\times t]+(1-F)\times \exp [-\,{k}_{2}\times t]]$$

Of course, if either *k*_1_ or *k*_2_ approach 0, and/or if *F* approaches 0 or 1, the BE model simplifies to the ME model.

The proposed gamma-exponential (GE) decay model is based on the following assumptions: (1) The radioactivity reduction processes, except physical decay, are summarized by the stretched exponential dependence *t*^*r*^, where *r* is an adjustable parameter. (2) The rate of these processes (*u*) follows a continuous probability distribution *P*(*u*). In other words, there is a continuous distribution of stretched exponential decay patterns. The GE model is mathematically represented as follows:

6 $${R}_{GE}(t)=\exp [Q]\times ({\int }_{u=0}^{\infty }\,\exp [\,-\,u\times {t}^{r}]\times P(u)\times du)\times PD(t)$$

The selected probability distribution *P*(*u*) is the following customized version of the Gamma distribution, where µ is an adjustable parameter (the mean), ν is a variance-determining parameter (so that variance = ν × µ^2^), and Γ is the Gamma function:

7 $$P(u)=({u}^{(\frac{1}{\nu }-1)}\times \exp \,[-\,\frac{u}{\mu \times \nu }])/({[\mu \times \nu ]}^{(\frac{1}{\nu })}\times {\rm{\Gamma }}(1/{\rm{\nu }}))$$

Explicit solutions to equations 6 and 7 on the linear and logarithmic scales are as follows:

8 $$\begin{array}{rcl}{R}_{GE}(t) & = & \exp [Q]\times PD(t)\times {(1+\mu \times \nu \times {t}^{r})}^{(-\frac{1}{\nu })},\\ \mathrm{ln}\,[{R}_{GE}(t)] & = & Q+\,\mathrm{ln}\,[PD(t)]-(1/\nu )\times \,\mathrm{ln}\,[1+\mu \times \nu \times {t}^{r}]\end{array}$$

When parameter ν approaches 0, the “width” of the *P*(*u*) distribution shrinks, and the GE model approaches stretched exponential behaviour where ln[*R*_*GE*_(*t*)] depends on −µ × *t*^*r*^. The same phenomenon occurs when µ approaches 0. When parameter *r* approaches 1, the GE model approaches mono-exponential behaviour and becomes identical to the ME model (equation 1) where µ is substituted for *k*.

The proposed power-decay (PD) model is based on the observation that deviations from simple exponential decay, such as the weighted sum of several exponential functions, can be parsimoniously represented by a power function like (1 + *z* × *t*)^−*u*^, where *z* and *u* are adjustable parameters. The resulting PD model equation is as follows, where *Q*, *P*(*u*) and *PD*(*t*) have the same meanings as in equations above:

9 $${R}_{PD}(t)=\exp [Q]\times ({\int }_{u=0}^{\infty }{(1+z\times t)}^{-u}\times P(u)\times du)\times PD(t)$$

Explicit solutions to equation 9 on the linear and logarithmic scales are as follows:

10 $$\begin{array}{rcl}{R}_{PD}(t) & = & \exp [Q]\times PD(t)\times {(1+\mu \times \nu \times \mathrm{ln}[1+z\times t])}^{(-\frac{1}{\nu })},\\ \mathrm{ln}\,[{R}_{PD}(t)] & = & Q+\,\mathrm{ln}\,[PD(t)]-(1/\nu )\times \,\mathrm{ln}\,[1+\mu \times \nu \times \,\mathrm{ln}\,[1+z\times t]]\end{array}$$

when parameter ν approaches 0, ln[*R*_*PD*_(*t*)] depends on −µ × ln[1 + *z* × *t*]. When parameter *z* approaches 0, the PD model approaches mono-exponential behaviour and becomes identical to the ME model (equation 1) where µ × *z* is substituted for *k*.

### Model fitting and parameter estimation

We used maximum likelihood estimation to find best-fit parameters for each of the 4 tested models (ME, BE, GE and PD) on each analysed data set. We maximized the following log likelihood function^16^, which assumes that the statistical uncertainties are normally distributed on a logarithmic scale, where N is the number of data points, σ is the statistical uncertainty magnitude parameter, *W*_*i*_ is the uncertainty “weight” for the *i*-th data point (so that the variance = [σ × *W*_*i*_]^2^), *R*_*O,i*_ is the observed radioactivity at the *i*-th data point, and *R*_*M,i*_ is the corresponding radioactivity predicted by the *M*-th model (i.e. by one of the tested models):

11 $$L{L}_{M}=-\,{\rm{N}}\times (\frac{\mathrm{ln}\,[2\times \pi ]}{2}-\,\mathrm{ln}[\sigma ])-\sum _{i=1}^{N}\,\mathrm{ln}[{W}_{i}]-\frac{1}{2\times {\sigma }^{2}}\times \sum _{i=1}^{N}\,{(\mathrm{ln}[{R}_{O,i}]-\mathrm{ln}[{R}_{M,i}])}^{2}/{{W}_{i}}^{2}$$

For all analysed data sets, expect for the wild boar data from Chernobyl (data set V)^15^, the *W*_*i*_ values were unknown and presumably equal, so we set them to 1 for all data points. For the Chernobyl boar data, the radioactivity measurements in individual animals were not reported^15^, so we used the reported summary data: number of animals (*n*_*i*_), mean (*AV*_*i*_) and maximum (*MAX*_*i*_) concentrations of ^137^Cs in boar muscle tissue (kBq/kg) at each *i*-th time point (during each studied year). These summary data were used to approximate ln[*R*_*O,i*_] and *W*_*i*_ as follows, where *GE*(*n*_*i*_) is the median extreme value for a sample of size *n*_*i*_ taken from the standard Normal distribution:

12 $$\mathrm{ln}\,[{R}_{O,i}]=\,\mathrm{ln}[A{V}_{i}],\,{W}_{i}=(\mathrm{ln}[MA{X}_{i}]\,-\,\mathrm{ln}[A{V}_{i}])/GE({n}_{i})$$

The values of *GE*(*n*_*i*_) were estimated from 30,000 Monte Carlo simulations using Maple 2017® software. For example, for *n*_*i*_ = 5 random values drawn from the standard Normal distribution, *GE*(*n*_*i*_) = 1.13, which means that the median value (across all simulations) for the maximum of the sample is approximately 1.13 standard deviation units above the mean. Of course, *GE*(*n*_*i*_) increases with *n*_*i*_, and for *n*_*i*_ = 20, *GE*(*n*_*i*_) reached 1.82. Here we used *GE*(*n*_*i*_) to approximate *W*_*i*_. Sensitivity calculations showed that the analysis results for this data set were not very sensitive to perturbations of *W*_*i*_.

The log likelihood function (equation 11) for each model on each data set was maximized using the sequential quadratic programming (SQP) algorithm in Maple 2017® software^17^. The following procedure was used to maximize the probability of finding the global, rather than a local, log likelihood maximum: (1) Initial parameter values for each model on each data set were identified manually by substituting various values and keeping those which generated a model curve that visually passed through most of the data. (2) Five hundred parameter values were randomly selected from the parameter space in the vicinity of the initial values. (3) The model was fitted using each of these 500 starting parameter combinations, and the combination which produced the highest log likelihood was retained as the best-fit parameter set. During the optimization procedure, the parameters were restricted to biologically plausible ranges, such as positive values or values > 10^−6^. Uncertainties (95% confidence intervals, CI) for each parameter were estimated using profile likelihood^18^.

### Information theoretic model selection

The performances of different models fitted to the same data were compared using the Akaike information criterion with sample size correction (AICc)^7,8^. AICc for the *M*-th model (AICc_M_) is calculated below, where Λ_M_ is the number of adjustable parameters and *LL*_M_ is the maximized log-likelihood value:

13 $$AIC{c}_{M}=-\,2\times L{L}_{M}+2\times {{\rm{\Lambda }}}_{M}+2\times {{\rm{\Lambda }}}_{M}\times ({{\rm{\Lambda }}}_{M}+1)/(N-{{\rm{\Lambda }}}_{M}-1)$$

The model that achieves the lowest AICc value is considered to be best supported among those considered. The relative likelihood of the *M*-th model is called the evidence ratio (ER_M_) and can be expressed as follows:

14 $$E{R}_{M}=\exp [-\,\frac{{\rm{\Delta }}AIC{c}_{M}}{2}],\,{\rm{\Delta }}AIC{c}_{M}=AIC{c}_{M}-AIC{c}_{min}$$

here, AICc_min_ is the lowest AICc value generated by the set of compared models.

The evidence ratio for *M*-th model, divided by the sum of the evidence ratios for all models, is the Akaike weight, *W*_M_. It represents the probability that the *M*-th model would be considered best-supported, among those tested, upon repeated sampling of the data. *W*_M_ is described by the following equation:

15 $${W}_{M}=E{R}_{M}/\sum _{M}\,\,E{R}_{M}$$

As noted above, the BE, GE and PD models can all approach simple exponential behaviour (i.e. simplify to the ME model) if certain parameters approach limiting values such as zero. Consequently, if any of these models failed to achieve a higher log likelihood than the ME model, their Akaike weights were assigned to the ME model. For example, if all 4 models had the same log likelihoods on a given data set, the simplest ME model was assigned an Akaike weight of 1 and all of the other more complex models were assigned weights of zero.

### Extrapolation of models from short to long times

On each data set, we compared how the different models performed when each model was fitted to the data at short times and then extrapolated to data at longer times. The comparison was performed by calculating root mean squared error (RMSE) for each model on each data set under the following conditions: (1) Fitting the *M*-th model to all the data over the entire available time range, but calculating RMSE only on 1/3 of the data at the longest times. This produced *RMSE*_*Full,M*_. (2) Fitting the *M*-th model only to those data points over the shortest 2/3 of the time range, but again calculating RMSE only on 1/3 of the data at the longest times. This produced *RMSE*_*Short,M*_. Based on these two metrics, we calculated the RMSE loss (*RMSEL*_*M*_), which represents a difference in RMSE between conditions 1 and 2:

16 $$RMSE{L}_{M}=RMS{E}_{Short,M}-RMS{E}_{Full,M}$$

Usually (but not always) *RMSEL*_*M*_ was a positive number, which suggests that RMSE calculated on data at long times was generally worse when the model was fitted to data at short times only instead of to all the data. The model that achieved the smallest RMSE loss, compared with other tested models, was considered to perform the best when extrapolated from short to long times. In other words, a small RMSE loss value indicates that the model has the right “shape” to decently predict the data at long times based on best-fit parameters obtained from fitting the data at short times. In addition, we also calculated RMSE for each model on each full data set.

## Results

### Comparison of discrete rate model and CPD model behaviours

Models that consist of sums of several discrete exponential rates are frequently used to analyse complex patterns of radioactivity excretion or retention in human and animal data. However, when a decay pattern involves two or more rates, models that assume a continuous distribution of rates, such as the GE and PD formalisms proposed here, can fit the data very similarly to discrete rate models, but using fewer adjustable parameters. For example, consider a sum of 3 exponential rates, where 60% of the radioactivity decays at a rate of 1 time^−1^, 30% decays at a slower rate of 1/16 time^−1^, and the remaining 10% decays at an even slower rate of 1/256 time^−1^. When GE and PD models were fitted by least squares (on a logarithmic scale) to the decay curve generated by this sum of three rates model, very similar curves were obtained (Fig. 1). Notably, the discrete-rates model contains six adjustable parameters (neglecting the statistical uncertainty term in this model comparison example), whereas the continuous rate distribution models contain two fewer parameters each (*Q* = 0, µ = 0.677 time^−1^, *r* = 0.606, ν = 0.857 for GE and *Q* = 0, µ = 0.588, *z* = 1.004 time^−1^, ν = 0 for PD, see Methods section for parameter meanings). This example illustrates the point that CPD models represent parsimonious alternatives to discrete rate models.

**Figure 2:**
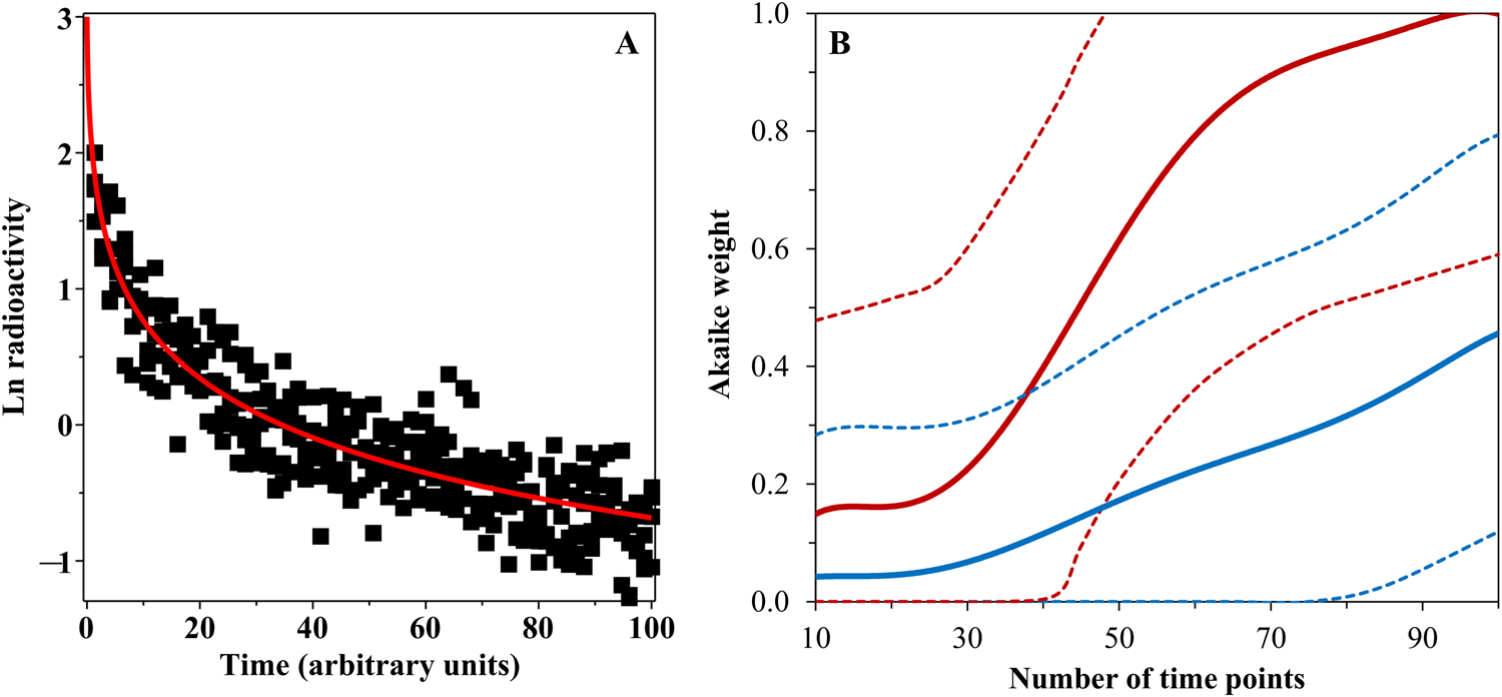
Information theoretic model selection examples on simulated data. (**A**) An example of data (black symbols) simulated from the GE model (red curve). (**B**) Summary of model selection results on simulated data sets with different numbers of time points. Blue solid curve = median Akaike weight for the GE model, which is the true data-generating model in this case. Blue dashed curves = 95% CIs. Red solid curve = median for the sum of Akaike weights for the GE and PD models. Red dashed curves = 95% CIs.

Because discrete rate and CPD models often produce similar fits to the data, relatively large and detailed data sets can be required to identify the best-supported model. We illustrate this point by simulating some data from a “true” GE model with parameters *Q* = 3.0, µ = 1.8 time^−1^, *r* = 2/3, ν = 1.0 (see Methods section for parameter meanings), and σ (the standard deviation) = 0.25 ln units. The simulations covered the time range of 0–100 arbitrary units, using 5 simulated data points per time point, but varying the number of regularly spaced time points from 10 to 100. For example, using 10 time points implies that the spacing between them would be 100/10 = 10 time units, and so the time points would be 10, 20, 30 and so on until 100. A sample simulation with 75 time points is shown in Fig. 2A. The simulated data were analysed by four models (ME, BE, GE and PD), using the information theoretic approach described in the Methods section. Although the GE formalism was the “true” data-generating model in this case, the median Akaike weight of the GE model across simulations was very low (<0.1) in data sets with <40 time points, and remained <0.5 even when 100 time points were used (Fig. 2B). Because the Akaike weights of all tested models have a sum of unity (see Methods section), such low values indicate that the GE model was usually not selected as best-supported on small data sets even though it was the true model. The median value for the sum of Akaike weights for the GE and PD models, however, increased more rapidly with increasing number of time points, and reached 0.5 at about 40 time points and 0.9 at about 70 time points (Fig. 2B). Therefore, this data simulation example shows that: (1) It can be quite difficult to identify the “true” data-generating model, or even any best-supported model, in radioactivity biokinetics data. (2) It is, however, feasible to discriminate between discrete rate models (ME and BE in this case) and continuous rate distribution models (GE and PD in this case) in sufficiently large data sets because summed Akaike weights for the GE and PD models grow rapidly with increasing number of time points (Fig. 2B). Thus, sufficiently detailed data should allow an investigator to estimate whether continuous rate distributions or discrete rates describe the studied process better by applying information theoretic criteria. This information would provide mechanistic insight and potentially increase the accuracy of model predictions.

### Analysis of data set I

**Figure 3:**
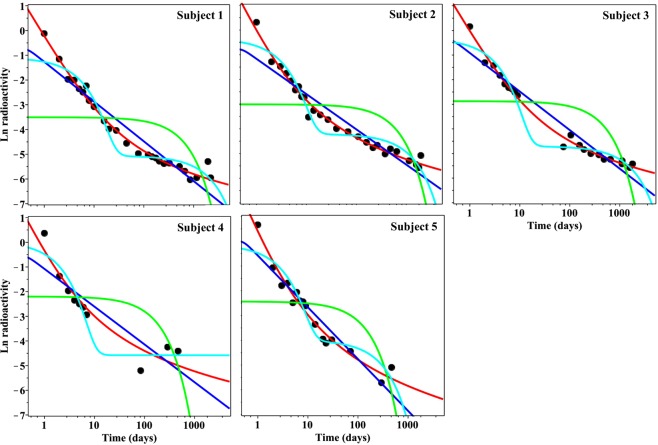
Model best fits to the human plutonium data (black circles), data set I. The y-axis represents ln-transformed urinary excretion of ^244^Pu by male volunteers after intravenous injection in citrate solution (%/day)^12^. The x-axis represents time after plutonium administration. Green curve = ME model, cyan curve = BE model, blue curve = GE model, red curve = PD model.

Our analysis of real data on plutonium excretion in human volunteers (data set I) showed that the PD model strongly outperformed all other tested models (ME, BE and GE) on data from 4 out of 5 subjects (Table 1, Fig. 3, Supplementary Data File 3). This conclusion was supported by the PD model’s very high Akaike weight (essentially unity) and low root mean squared error (RMSE), compared with other models (Supplementary Data File 3). The PD model also outperformed other models when each model was fitted to data at short times and extrapolated to longer times. This is shown by the PD model’s low RMSE loss value (see Methods section), compared with other models (Supplementary Data File 3).

Subject 4 was an exception: the BE model was best-supported, whereas the PD model and the PD and GE models taken together had much lower Akaike weights (Table 1, Supplementary Data File 3). However, the best RMSE loss was still achieved by the PD model (Table 1, Supplementary Data File 3). The likely reason for why the analysis results for subject 4 differed from those for the other subjects is the absence of data points at times between 7 and 84 days (Fig. 3).

Overall, these results suggest that the PD formalism, which assumes a continuous distribution of decay rates, reasonably approximated most of the plutonium data in a parsimonious manner, outperforming other tested models. The PD model (and the other formalisms tested here) are intended to generate simple and parsimonious “summaries” of the complex processes of plutonium biokinetics in the human body processes using a minimum number of parameters. In contrast, there is a different class of models - detailed and highly parametrized mechanistic formalisms, such as the model by Leggett *et al*.^1^. We believe that detailed biokinetics models and the simple summary models proposed here have different purposes. Both model classes can be applied to plutonium data, depending on the goals of the investigator.

### Analysis of data set II

**Figure 4:**
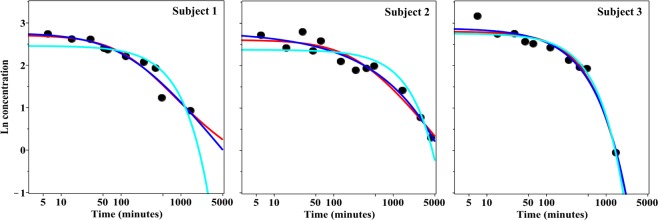
Model best fits to the human strontium data (black circles), data set II. The y-axis represents ln-transformed plasma concentrations (µg/L) of ^84^Sr and ^86^Sr for three individuals, as a function of time after administration^13^. The x-axis represents time after strontium administration. Green curve = ME model, cyan curve = BE model, blue curve = GE model, red curve = PD model.

A different pattern was seen in the analysis of data set II on strontium retention in healthy human volunteers (Table 1, Fig. 4, Supplementary Data File 3). The decay kinetics appeared simpler than the kinetics for plutonium, possibly because of smaller data set size and shorter investigated time range. The ME model was best-supported on data for 2 out of the 3 subjects, but continuous rate distribution models achieved the best RMSE loss on data from all 3 subjects (Table 1, Supplementary Data File 3). These results suggest that strontium retention in the human body, perhaps expectedly, may deviate somewhat from ME kinetics, but that this deviation is difficult to quantify using the data and models described here.

### Analysis of data set III

**Figure 5:**
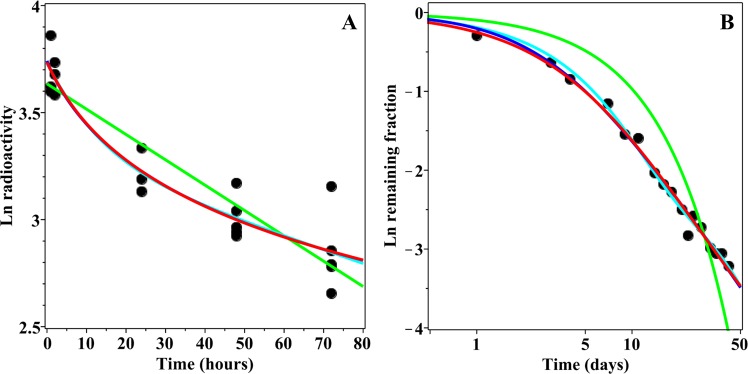
Model fits to data set III: laboratory animal data. (**A**) Model best fits to the data on melanin-binding ^111^In-labeled antibody in mouse blood (black circles). The y-axis represents percentage of injected radioactive dose per gram tissue, corrected for physical decay of ^111^In and ln-transformed. The x-axis represents time after injection. (**B**) Model best fits to radiocaesium data in sea urchins (black circles). The y-axis represents the ^137^Cs radioactivity concentration in the sea urchins^14^, relative to its concentration in water, divided by the value at day 0 and ln-transformed. The x-axis represents time after the urchins were placed in non-radioactive water. In both panels, green curve = ME model, cyan curve = BE model, blue curve = GE model, red curve = PD model.

Data set III contains two examples of radioactivity retention kinetics in animals measured under laboratory conditions. The first example, melanin-binding ^111^In-labeled antibodies in mouse blood, was visually well described by the BE, GE and PD models (Fig. 5A), but because of the data set’s small size the simplest ME model achieved the highest Akaike weight (Supplementary Data File 3). The Akaike weights and RMSE values showed little difference between different models (Supplementary Data File 3 online), suggesting that none of these models clearly outperformed the others. RMSE loss was lowest for the PD model, which provides some indication that this formalism performed better than others when extrapolated from short to long times on this data set (Table 1, Supplementary Data File 3).

The second example, radiocaesium retention in sea urchins, appeared to be best described by the BE model (Table 1, Supplementary Data File 3), as reported in the original publication from which data were extracted^14^. The GE and PD models, however, were not far behind the BE model, the sum of Akaike weights for the GE and PD models was >0.5 (Table 1), and all three formalisms produced similar best-fit curves (Fig. 5B). The BE, GE and PD models all had similar RMSE values, and the PD model had the best RMSE loss performance (Table 1, Supplementary Data File 3). The ME model had very poor support (Supplementary Data File 3). These results suggest that radiocaesium biokinetics in sea urchins involves at least two, and possibly more, decay rates. These rates are relatively rapid, which is consistent with observations that radiocaesium levels in sea urchins after the Fukushima nuclear disaster declined to undetectable levels within 3–4 years after the accident, whereas they remained elevated for much longer time in some other marine organisms like bottom-dwelling fish^19^.

### Analysis of data set IV

**Figure 6:**
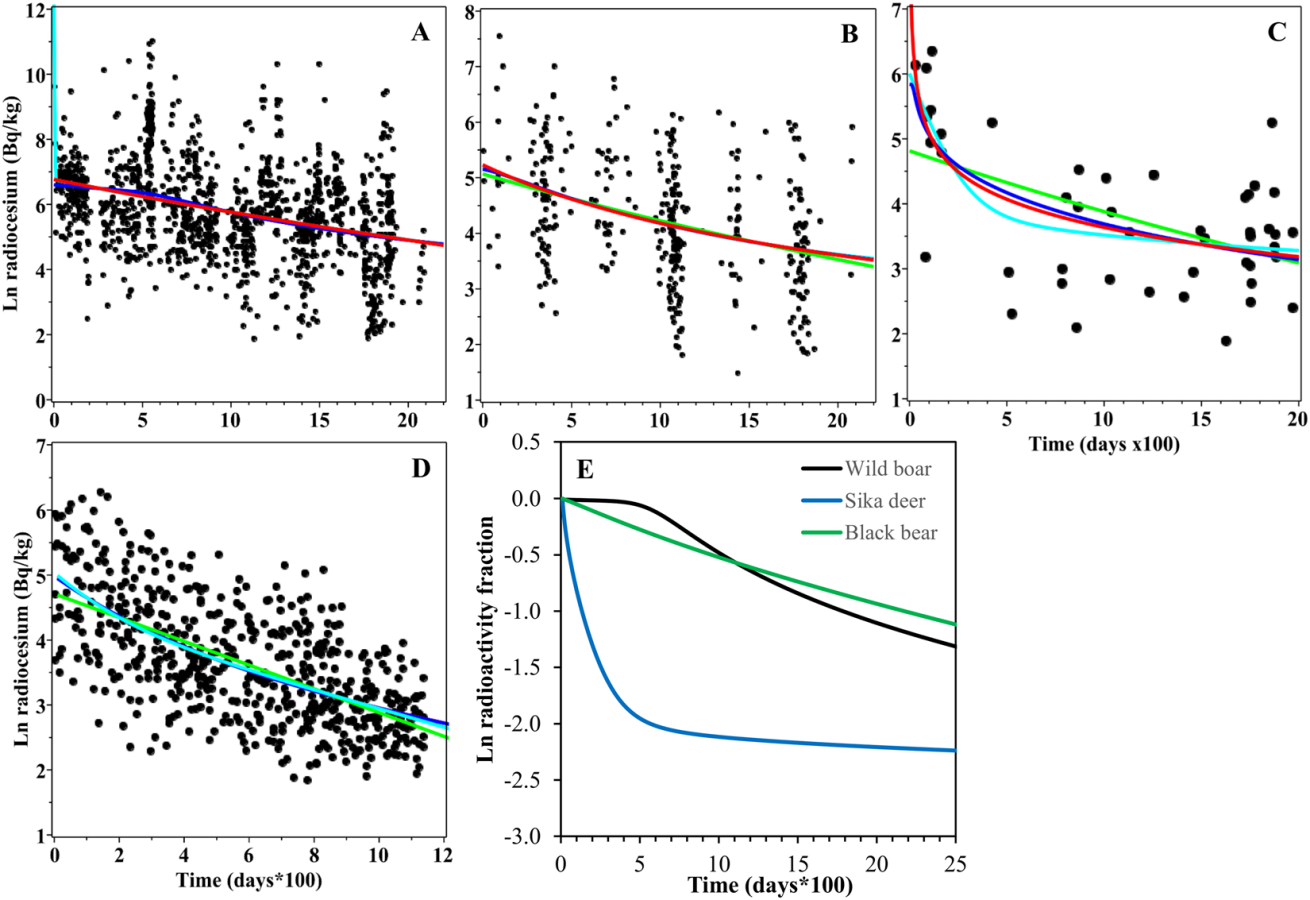
Model fits to data set IV: radiocaesium levels in animals from areas contaminated by the Fukushima nuclear accident. Panels (A–C) show the time kinetics of radioactivity levels in different species. (**A**) wild boars, (**B**) black bears, (**C**) sika deer, (**D**) ocellate spot skate. Black symbols = data points, green curve = ME model, cyan curve = BE model, blue curve = GE model, red curve = PD model. Panel € shows the radioactivity time kinetics in the three terrestrial animal species (boars, bears and deer), but normalized to the values at time zero. The curves represent weighted averages of all four models (ME, BE, GE and PD) constructed by MMI. Black curve = wild boars, green curve = black bears, green curve = sika deer.

Data set IV contains four examples of ecological radioactivity data in terrestrial and aquatic animals in regions contaminated by the Fukushima nuclear power plant accident in Japan. On the first example, radiocaesium data in wild boars, the PD model simplified to approach the ME model, and the BE model exhibited unreasonable behaviour at short times (Table 1, Fig. 6A, Supplementary Data File 3). The GE model strongly outperformed all other tested formalisms (Table 1, Supplementary Data File 3), probably because it was the only model flexible enough to reproduce a slow decay pattern at short times, followed by faster decay at longer times, and the large data set size allowed this difference in curve shapes to become important (Fig. 6A). These results suggest that the decrease in radiocaesium concentrations in wild boars after the Fukushima nuclear power plant accident is probably influenced by multiple ecological factors that can be approximated by the GE model, but not by the other tested models such as those that assume only one or two decay rates.

In contrast, radiocaesium in black bears from the same contaminated area of Japan appeared to decrease over time with simpler kinetics than in wild boars: the ME model achieved the highest support on black bear data (Table 1, Supplementary Data File 3). The best fits from all other models essentially overlapped the one from the ME model (Fig. 6B), and the ME model was favoured in information theoretic analysis due to its simplicity. Sika deer data from the Fukushima-contaminated area displayed yet another pattern: they were best described by the BE model, which predicted rapid decay at short times and essentially no decay (other than physical) at longer times (Table 1, Fig. 6C, Supplementary Data File 3).

Model-averaged (MMI) best-fit curves for boars, bears and deer are shown in Fig. 6E. These species living in a contaminated habitat were subjected to continuous intakes of radionuclides and hence approached a semi-equilibrium in terms of radionuclide intake and excretion, which is often attained after a year or two after the initial contamination event. The time-patterns seen here therefore primarily reflect ecological processes, rather than the metabolic patterns in the species.

The last example, aquatic ocellate spot skate data, favoured the BE model, although the sum of Akaike weights for the GE and PD models was >0.5 (Table 1, Fig. 6D, Supplementary Data File 3). Most of the tested models produced visually similar fits to the data, and their RMSE and RMSE loss values were similar (Table 1, Fig. 6D, Supplementary Data File 3). The ocellate spot skate is an example of a bottom-dwelling fish species in which radiocaesium levels remained elevated even several years after the Fukushima accident^19^.

### Analysis of data set V

**Figure 7:**
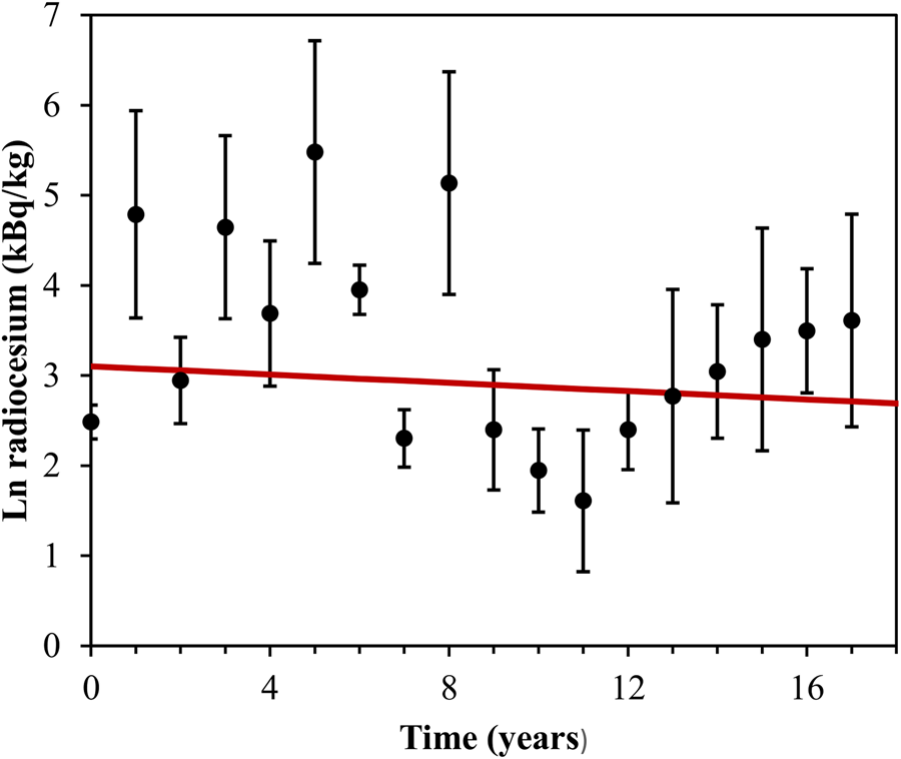
Model best fits to the radiocaesium data in Chernobyl wild boars (data set V). Black circles represent data points, error bars represent standard deviations. The y-axis represents the ln-transformed ^137^Cs radioactivity concentration in boar meat^15^. The x-axis represents time after 1991. The fits from all models overlapped on this data set. They are shown by the same red curve.

Our analysis of the final data set (V) on ^137^Cs in wild boars from the area contaminated by the Chernobyl nuclear power plant accident^15^ suggested that caesium levels in Chernobyl boars (Table 1, Fig. 7) did not decrease at all due to ecological processes over the studied period. The best-fit curves from all tested models therefore overlapped, with the decrease in caesium radioactivity attributable exclusively to physical decay (Fig. 7). This pattern likely reflects the ecological turnover of radiocaesium and the dietary habits of the studied species. Wild boars consume mushrooms, which strongly accumulate caesium^20^, and the boar radioactivity fluctuations over the years may arise from the fact that particular years exhibit a more intense mushroom growth.

## Discussion

We applied two proposed models with continuous probability distributions of decay rates (GE and PD) and two conventional models with sums of discrete decay rates (ME and BE) to a deliberately diverse collection of human and animal data sets on the biokinetics of radioactive materials in living organisms. This collection included kinetics data on multiple elements (plutonium, strontium, caesium, indium) generated in the laboratory (data sets I-III) and under natural conditions in areas contaminated by nuclear accidents (data sets IV-V). The focus of the study was to evaluate how the selected assemblage of four models behaves on each of these different example data sets, and what inferences these models can provide about the biological, chemical and ecological processes involved in radionuclide kinetics.

The rationale for developing and using these models was that simple formalisms can often bring out the key features of the studied system and produce reliable interpolations and extrapolations, based even on limited amounts of data, using only a few adjustable parameters^3,4,21^. In the case of radionuclide biokinetics, simple easy to use models are needed for predicting radioactivity values at long times based on data measured at shorter times. Such predictions are important in many situations, e.g. for estimating the cumulative radiation dose absorbed by a person exposed to radionuclides in a medical setting, or for estimating how long after a nuclear accident will game animals in the area retain radioactivity concentrations too high for human consumption.

The most common approach to simple modelling of radionuclide biokinetics involves a sum of exponential decay rates^22–26^. A certain fraction of radioactivity (e.g. the one contained in a given organ or body “compartment”) is assumed to be eliminated at one rate, another fraction at a different (e.g. slower) rate, and so on. In this system, the different rate constants have compensatory effects: a decrease in one rate can be compensated for by increases in other rate(s). Consequently, parameter uncertainties tend to be large^4^ and extrapolation of model predictions to times longer than those used for model fitting tends to be unreliable.

These problems can be addressed by assuming that the complex chemical, biological and ecological processes during radionuclide excretion or retention can be approximated by a continuous distribution of decay rates. In other words, instead of using a sum of several discrete rates, one can use an integral over an infinite number of rates described by a certain probability distribution. This approach, which is known in pharmacokinetics, chemistry and physics^3,6,21,27–29^, allows multiple incompletely understood processes to be combined into a “mixture” represented by only a few adjustable parameters. Specifically, solutions for kinetic equations that characterize relaxation in disordered systems often lead to simple functions such as the stretched exponential or stretched hyperbola, which are known since the 19^th^ century^5,6,21,29^.

We used this line of reasoning to develop the GE and PD models. The diverse data sets chosen for model testing were intended to represent a variety of scenarios where modelling of radionuclide biokinetics can be important. They also represent a variety of decay patterns, from simple to more complex. We fitted each model to each data set and compared model performances by information theoretic approaches^7,8^. We also compared the ability of each model on each data set to extrapolate from short times to longer times.

As expected, the relative performances of different models varied widely depending on data set. The variability in model performance on different data sets likely reflects underlying differences in radionuclide biokinetics due to differences in organism, radionuclide type and environment, and the effects of data set size and structure. Whereas on some data sets one model was dominant (e.g. the PD model on data set I), in other cases no model was strongly favoured over others (e.g. on data set III). On one data set (V) none of the models was required to explain the data because the time trend was sufficiently explained by physical radionuclide decay alone. Larger data sets (e.g. IV, I) of course tend to support more complex models and provide more information for model selection, than smaller ones (e.g. III, VII).

The sum of Akaike weights for the proposed GE and PD models can be interpreted as evidence for >2 decay rates being involved. This sum was frequently >0.5 among the human and animal data sets analysed here (Table 1). Even more frequently, the GE and/or PD models achieved the lowest RMSE loss, suggesting that these models tend to be more robust than discrete rate models (ME and BE) during extrapolation from short to long times (Table 1). Even greater robustness can potentially be achieved by model averaging (MMI), particularly when no single model has a clearly dominant Akaike weight.

Taken together, our results suggest that radionuclide biokinetics in living organisms are often sufficiently complex to be reasonably described by a continuous distribution of decay rates (as assumed in the GE and PD models), than by the commonly used assumption of one or two discrete rates (as in the ME and BE models). For example, the PD model showed strong performance on human plutonium excretion data and is notably much more parsimonious than the extensively parametrized multi-compartment kinetic models^30,31^. Consequently, we think that CPD models are useful alternatives to discrete rate models.

The CPD approach can be used to determine whether the radionuclide biokinetics involve multiple compartments and/or rate constants. Robust results based on all tested models can be obtained using MMI^7,8^, and they can prove to be very useful in many situations involving medical use of radionuclides and accidental or malicious radioactive contamination.

## Supplementary information

Supplementary Dataset 1

Supplementary Dataset 2

Supplementary Dataset 3
